# Protein Kinase D1 attenuates tumorigenesis in colon cancer by modulating β-catenin/T cell factor activity

**DOI:** 10.18632/oncotarget.2277

**Published:** 2014-08-04

**Authors:** Vasudha Sundram, Aditya Ganju, Joshua E. Hughes, Sheema Khan, Subhash C. Chauhan, Meena Jaggi

**Affiliations:** ^1^ Cancer Biology Research Center, Sanford Research/USD, Sioux Falls, SD, USA; ^2^ Department of Pharmaceutical Sciences and Center for Cancer Research, University of Tennessee Health Science Center, Memphis, Tennessee

**Keywords:** Colon Cancer, PKD1, β-catenin, T cell factor activity (TCF), Tumor suppressor, Cell motility, Cell invasion

## Abstract

Over 80% of colon cancer development and progression is a result of the dysregulation of β-catenin signaling pathway. Herein, for the first time, we demonstrate that a serine-threonine kinase, Protein Kinase D1 (PKD1), modulates the functions of β-catenin to suppress colon cancer growth. Analysis of normal and colon cancer tissues reveals downregulation of PKD1 expression in advanced stages of colon cancer and its co-localization with β-catenin in the colon crypts. This PKD1 downregulation corresponds with the aberrant expression and nuclear localization of β-catenin. *In-vitro* investigation of the PKD1-β-catenin interaction in colon cancer cells reveal that PKD1 overexpression suppresses cell proliferation and clonogenic potential and enhances cell-cell aggregation. We demonstrate that PKD1 directly interacts with β-catenin and attenuates β-catenin transcriptional activity by decreasing nuclear β-catenin levels. Additionally, we show that inhibition of nuclear β-catenin transcriptional activity is predominantly influenced by nucleus targeted PKD1. This subcellular modulation of β-catenin results in enhanced membrane localization of β-catenin and thereby increases cell-cell adhesion. Studies in a xenograft mouse model indicate that PKD1 overexpression delayed tumor appearance, enhanced necrosis and lowered tumor hypoxia. Overall, our results demonstrate a putative tumor-suppressor function of PKD1 in colon tumorigenesis *via* modulation of β-catenin functions in cells.

## INTRODUCTION

Colorectal cancer is the third most commonly diagnosed cancer and the second leading cause of cancer death in the US with approximately 51,000 deaths per year [1]. It often begins as a benign polyp in the colon, which over time may become cancerous. The deregulation of the β-catenin signaling pathway due to mutations in the *APC-Axin* or *β-catenin* genes is correlated with over 80% of colon cancer [2]. Therefore, understanding the expression, localization and regulation of β-catenin protein and modulation of β-catenin signaling pathway function is critical for developing novel strategies for treatment and/or preventing of colon cancer.

Studies have identified that inhibitors of the PTEN/Akt/GSK3β signaling cascade and regulation of β-catenin act as potential agents to effectively target cancer stem cells and tumorigenic cancer cells [3, 4]. β-catenin is a highly conserved, bi-functional protein that functions as a transcription factor in the Wnt signaling pathway to regulate cell proliferation and differentiation [5, 6]. In addition, at the cell membrane, it plays a key role in regulating E-cadherin mediated cell-cell adhesion by binding to and anchoring E-cadherin to the actin cytoskeleton through the adaptor protein, α-catenin. In the absence of Wnt-signaling, β-catenin is primarily bound to cadherin and the N-terminus of free cytosolic β-catenin is targeted for phosphorylation, ubiquitination and degradation by APC-Axin-GSK3β-CK1 complex. β-catenin is also phosphorylated at other sites by the diverse kinases PKA, AKT, and JNK2 that promotes β-catenin activity and its nuclear translocation [7]. Mutations in APC, Axin, or these N-terminal phosphorylation sites of β-catenin are found in multiple types of human cancers, where these mutations elevate β-catenin posttranscriptional stability, signaling [8] and formation of nuclear β-catenin/TCF complexes [9]. In these scenarios, β-catenin localizes to the nucleus and enhances the transcription of proto-oncogenes such as c-Myc, c-Jun and Cyclin D1, resulting in initiation and progression of cancer [5, 6].

Protein Kinase D1 (PKD1) is a ubiquitously expressed serine/threonine kinase that plays a key role in several signal-transduction pathways [10-12] through regulatory domains that are homologous to the PKC family and the presence of functional kinase domain with substrate specificity homologous to those of the CaMK family [10]. Therefore, PKD1 has been found to modulate a number of cellular processes including cell proliferation, cellular motility, invasion, aggregation and epithelial-mesenchymal transition [13-21]. Downregulation of PKD1 has been documented in breast and prostate cancers [10, 13, 20, 22]. In breast cancer, epigenetic silencing of *PRKD1* gene promoter has been reported to directly correlate with the loss of PKD1 expression and the invasive potential of breast tumors or cells [22]. Suppression of PKD1 expression was found to be associated with enhanced cellular invasion *via* modulation of multiple matrix metalloproteinases (MMPs) in breast cancer cells [13]. Previous work from our group has implicated an important role for PKD1 in prostate cancer [19-21] including modulation of E-cadherin, β-catenin functions, and androgen receptor signaling pathways [15, 21, 23-26]. Herein, we have investigated the role of PKD1 in colon cancer. We examined the staining pattern of PKD1 expression in tissue of normal colon and colon cancer and demonstrated that PKD1 co-localized with β-catenin in normal colon tissues. In addition, PKD1 expression was downregulated in colon cancer tissues and this coincides with a corresponding change in the subcellular localization of β-catenin. For *in-vitro* analyses, we used SW480 and SW48 colon cancer cell lines to investigate and evaluate the effect of PKD1 overexpression on cellular characteristics. *In-vitro* and *in-vivo* studies using xenograft mouse model revealed that PKD1 overexpression suppresses cell proliferation, clonogenic potential, enhances cell-cell aggregation and alters the tumor histo-architecture *via* modulation of β-catenin functions in cells.

## RESULTS

### PKD1 is downregulated in colon cancer

**Figure 1:**
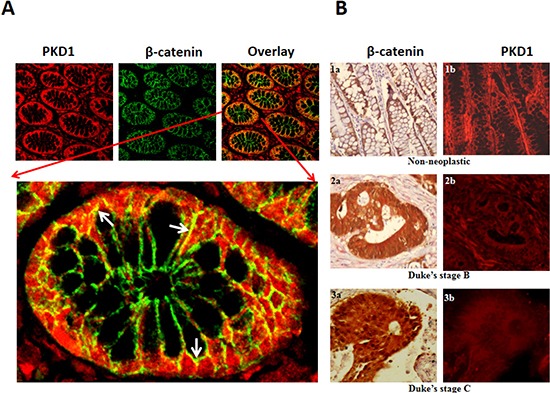
Expression of PKD1 is downregulated in colon cancer **(A)** *Immunohistochemical analysis of PKD1 and β-catenin in colon tissues*: Normal colon tissues were immunostained for PKD1 (red) and β-catenin (green). PKD1 expression was detected in the cytoplasm and membrane of colon cells, while β-catenin was primarily localized to the membrane. Co-localization of PKD1 with β-catenin was also detected (yellow). A magnified image of a single colon gland is shown to demonstrate co-localization of PKD1 and β-catenin (white arrows). Original magnification 200X. **(B)** *Tissue microarray (TMA)*: Colon cancer TMA slides were stained for β-catenin (brown) and PKD1 (red). β-catenin staining revealed distinct change in subcellular localization in colon cancer. It was primarily localized on the membrane of non-neoplastic samples (1a), while distinct cytoplasmic and prominent nuclear staining was detected in Duke's stage B (2a) and Duke's stage C colon cancer, respectively (3a). PKD1 expression was strongly detected in non-neoplastic samples (1b, red). However, PKD1 was progressively downregulated in Duke's stage B (2b) and Duke's stage C (3b) colon cancer. Original magnification 400X.

The deregulation of PKD1 expression is associated with various cancers including prostate and breast cancer [10, 13, 19, 20]. However, the expression profile of PKD1 in colon cancer is not known. Therefore, we investigated the expression pattern of PKD1 by immunofluorescence staining of colon tissues using anti-PKD1 antibody and fluorescently labeled secondary antibodies (red) (Figure 1A). Additionally, tissues were also simultaneously co-stained for β-catenin expression using anti-β-catenin antibody. Representative images from normal colon tissue stained for PKD1 and β-catenin are shown in Figure 1A. PKD1 expression (red staining) was predominantly detected in the cytoplasm with some expression on the membrane and in the nucleus of the cells, while β-catenin expression (green staining) was primarily localized to the membrane of the cells. The immunohistochemical (IHC) staining also revealed co-localization of PKD1 and β-catenin in colon tissues (Figure 1A, lower panel). This suggests a role for PKD1-β-catenin interaction in colon tissues. In order to investigate the expression profile of PKD1 in colon cancer tissues and quantitatively analyze changes in PKD1 or β-catenin expression, IHC analysis was performed on tissue microarray (TMA) slides containing normal (n=8) and colon cancer tissues (n=45) using chromogenic dyes (Figure 1B). The tissue samples were grouped based on the Dukes' staging of colon cancer into non-neoplastic, Duke's stage B (wherein the cancer has invaded the bowel walls, but has not spread to the lymph nodes) and Duke's stage C colon cancer (wherein the cancer has spread to the nearby lymph node) and analyzed for the levels of expression and the localization pattern of the proteins. PKD1 expression was significantly (p<0.05) downregulated in the cancerous tissues compared to normal tissues (Figure S1A). We also detected a trend in the progressive downregulation of PKD1 expression from non-neoplastic stage to Duke's stage B and Duke's stage C colon cancer (Figure 1B). The suppression of PKD1 expression coincided with the distinct change in the β-catenin localization in cancer tissues. While β-catenin was primarily localized on the membrane of normal colon glandular cells, a higher β-catenin staining was detected in the cytoplasm and the nucleus as the cancer progressed from Duke's stage B to Duke's stage C colon cancer, when the cancer had spread to the nearby lymph node. The association between downregulation of PKD1 expression with the change in β-catenin localization in colon cancer seems to suggest a role for PKD1 in regulating β-catenin functions in colon cancer. Based on these results, we proposed that PKD1 functions as a tumor suppressor *via* modulating β-catenin signaling pathway and inhibiting nuclear β-catenin function to suppress colon cancer growth. Thus an increase in PKD1 levels in colon cancer cells can inhibit the progression of colon cancer.

### Exogenous expression of PKD1

In order to investigate the functional role of PKD1 and the importance of PKD1-β-catenin interaction in colon cancer, we sought to overexpress PKD1 in a cell line that expresses low or no PKD1 and express high amounts of nuclear β-catenin, to mimic advanced stage colon cancer. Therefore, we screened a panel of five colon cancer cell lines for PKD1 expression using immunoblotting techniques. Moderate level of PKD1 expression was detected in almost all the cancer cell lines (SW480, SW48, T-84 and LoVo), except the HT-29 cell line, which showed very little PKD1 expression (Figure S1B). These cell lines were also analyzed by confocal microscopy to determine the expression pattern of β-catenin in cells. The SW480 cells primarily expressed β-catenin in the nucleus (Figure S1C). This is clearly evident by the appearance of the yellow color in the overlay image between β-catenin staining (green) and nuclear staining (red). The other remaining cells lines revealed a predominant cytoplasmic staining of β-catenin (data not shown). Therefore, we used the SW480 colon cancer cells to stably overexpress PKD1 and analyze its role in the regulation of nuclear β-catenin activity and colon carcinogenesis. Actively growing SW480 cells were chemically transfected with either GFP tagged PKD1 (pEGFP-PKD1) or control GFP vector (pEGFP) and subjected to fluorescence assisted cell sorting to enrich a pool of SW480 cells overexpressing either GFP tagged PKD1 or GFP. Over 60% of the stably transfected SW480 cells overexpressed our protein of interest (Figure S1D). Analysis of protein lysates from these cells by immunoblotting using anti-PKD1 antibody (Figure S1E) also revealed the overexpression of GFP tagged PKD1 in addition to endogenous PKD1 in the PKD1 overexpressing cells. For ease of description, from here onwards, the SW480 cells overexpressing GFP tagged PKD1 will be referred to as SW480-PKD1-GFP and the control cells overexpressing GFP vector will be referred as SW480-GFP.

### Exogenous expression of PKD1 inhibits cell proliferation

**Figure 2:**
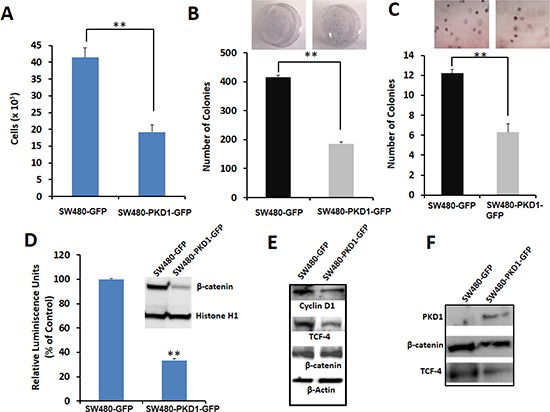
PKD1 overexpression decreases tumorigenic phenotypes by inhibiting the nuclear transcriptional activity of β-catenin in SW480 colon cancer cells **(A)** *Cell proliferation*. An equal number of SW480-GFP and SW480-PKD1-GFP cells were plated and after 48h the amount of ATP present in the metabolically active cells was measured using CellTiter-Glo Reagent. PKD1 overexpression decreased cell proliferation by over 50%. Mean ± SE; n=3; **p<0.05. **(B)** *Anchorage dependent colony formation*. SW480 cells overexpressing either PKD1 or GFP vector (2×10^3^) were plated in 100mm dishes for 12 days and the average number of colonies formed was counted and graphed. PKD1 overexpression inhibited anchorage dependent colony formation in SW480 cells. Mean ± SE; n=3; **p<0.05. **(C)** *Anchorage independent colony formation*. SW480-GFP and SW480-PKD1-GFP cells (4×10^4^) were seeded in 0.3% agarose and grown for 14 days. The number of colonies formed was enumerated and plotted. PKD1 overexpression decreased anchorage independent colony formation in SW480 cells. Mean ± SE; n=3; **p<0.05. **(D)** *Effect of PKD1 overexpression on β-catenin transcription activity*. Reporter luciferase assay was used to measure β-catenin transcription activity. The SW480-PKD1-GFP and SW480-GFP cells were transiently transfected with either a TCF-promoter-luciferase construct or a mutant TCF-promoter luciferase construct along with an internal control plasmid expressing *Renilla* luciferase gene. The β-catenin transcription activity was measured, normalized to the control *Renilla* luciferase activity and expressed as a ratio of TCF-promoter-luciferase activity to mutant TCF-promoter luciferase activity. The β-catenin activity of control SW480-GFP cells was set to 100%. PKD1 overexpression decreased the β-catenin transcription activity by over 70%. The inset depicts representative blots of nuclear lysates isolated from SW480-GFP or SW480-PKD1-GFP cells and probed for β-catenin expression. Histone H1 was used as internal control. PKD1 overexpression decreased nuclear β-catenin expression in SW480 cells. **(E)** *Effect on downstream targets*. Total protein isolated from SW480-PKD1-GFP or control SW480-GFP cells was resolved on gel and immunoblotted using specific antibodies. β-actin was used as loading control. PKD1 overexpression decreased Cyclin D1 and TCF-4 levels, both of which are downstream products of β-catenin/TCF transcription activity. **(F)** *Immunoprecipitation (IP)*. Equal amounts of nuclear extract isolated from the PKD1 overexpressing cells or control cells were subjected to IP using anti-TCF-4 antibody. The immune-complexes were resolved on gel and sequentially probed for β-catenin, TCF-4 and PKD1. PKD1 overexpression decreased the amount of TCF-4/β-catenin complex in the nucleus.

The stable SW480-PKD1-GFP and control SW480-GFP cells were examined for the effect of PKD1 overexpression on tumorigenic characteristics like cell proliferation and colony formation. PKD1 overexpression significantly (p<0.05) decreased cell proliferation compared to control SW480-GFP cells (Figure 2A). We next examined the clonogenic potential of these cells, an important parameter that reflects the ability of single cancer cells to survive, grow and colonize. PKD1 overexpression (SW480-PKD1-GFP) significantly reduced the ability of colon cancer cells to form anchorage dependent colonies, compared to control cells (Figure 2B). The anchorage independent clonogenic assay attempts to mimic the *in-vivo* situation and evaluates the ability of cells to form independent colonies when suspended in a gel or viscous medium in the absence of any anchor. Similar to results observed in anchorage dependent assay, SW480-PKD1-GFP cells formed fewer number of colonies compared to control SW480-GFP cells in anchorage independent assay. These results indicate a tumor suppressor function for PKD1 in colon cancer.

To ensure these results were not specific to one cancer cell line, a different colon cancer cell line, SW48, was also used to overexpress PKD1 or GFP and examine the effect on cell proliferation. This cell line was chosen since SW48 cells express a relatively low amount of PKD1 protein and is amenable to assess nuclear β-catenin transcription activity. In addition, unlike the SW480 cells, SW48 cells do not harbor any mutation in the *APC* gene which plays vital role in the regulation of β-catenin levels within the cells. Therefore, the SW48 cells were transiently transfected to overexpress PKD1 or GFP and analyzed for cell proliferation and clonogenic potential (Figure S2). Fluorescent and phase contrast image of the cells showed over 70% expression of the exogenous proteins (Figure S2A). Analysis of cell proliferation revealed that PKD1 overexpression significantly (p<0.05) decreased cell proliferation of SW48 cells, compared to control SW48-GFP cells (Figure S2B). PKD1 overexpression also significantly decreased both anchorage dependent and anchorage independent clonogenic potential of SW48 cells (Figure S2C and S2D) indicating that the anti-carcinogenic functions of PKD1 in colon cancer were a cell line independent phenomenon.

### PKD1 overexpression modulates β-catenin functions and subcellular localization

Dysregulation of β-catenin expression or functions leads to enhanced carcinogenesis by up-regulating the expression of various proto-oncogenes, thereby increasing cell proliferation, survival, motility, invasion and epithelial-mesenchymal transition (EMT) [2, 5, 10]. To investigate the underlying mechanism responsible for the anti-proliferative potential of PKD1 and given that PKD1 co-localized with β-catenin in colon tissues, we used a reporter assay to analyze the effect of PKD1 overexpression on the co-transcription activity of β-catenin. PKD1 overexpression significantly (p<0.05) downregulated β-catenin co-transcription activity by over 60% compared to control cells (Figure 2D). Decrease in the β-catenin co-transcription activity was likely a result of lower nuclear β-catenin levels. This is suggested by finding the decreased β-catenin expression in the nucleus on PKD1 overexpression (inset of Figure 2D). Additionally, we observed that PKD1 overexpression substantially decreased Cyclin D1 (downstream target of β-catenin) and TCF-4 expression (that is regulated by TCF-4/β-catenin) in SW480-PKD1-GFP cells compared to control cells. However, no change in the overall expression of β-catenin was observed in PKD1 overexpressing cells compared to control.

In order to detect complex formation between nuclear β-catenin, TCF-4 and PKD1, equal amounts of protein extracted from the nuclear lysates of SW480-PKD1-GFP or SW480-GFP cells were subjected to immuno-precipitation using anti-TCF-4 antibody (Figure 2F). The immuno-precipitated complex was resolved on a gel, blotted on a membrane and probed using specific antibodies against β-catenin, PKD1 and TCF-4. A lower level of TCF-4 and β-catenin and therefore lower β-catenin/TCF-4 transcription complex was detected in the PKD1 overexpressing cells compared to the control cells (Figure 2F). This result indicates that the lower β-catenin co-transcription activity detected in the PKD1 overexpressing cells was a consequence of a decrease in nuclear TCF-4/β-catenin complex in the PKD1 overexpressing cells compared to GFP control cells. The effect of PKD1 overexpression in attenuating β-catenin transcription activity was also detected in SW48 colon cancer cells. In these cells PKD1 overexpression decreased β-catenin transcription activity by over four folds (Figure S2E). These data suggest a critical role of PKD1 in the regulation of nuclear β-catenin transcription activity.

### Enzymatically functional kinase activity of PKD1 is required for the suppression of nuclear β-catenin transcription

**Figure 3:**
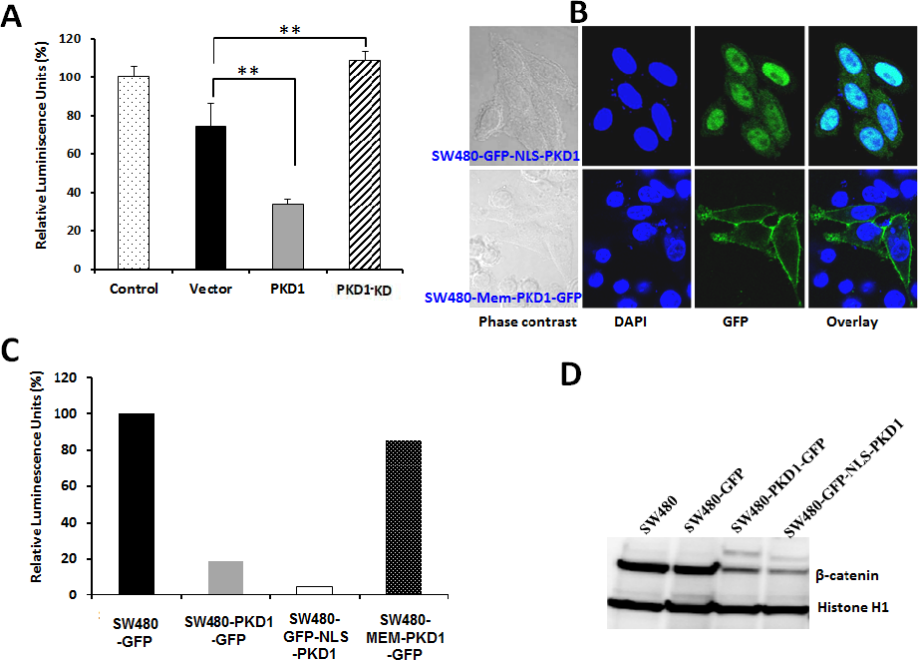
Enzymatically active PKD1 is required for decreasing β-catenin co-transcription activity **(A)** *Effect of kinase-dead PKD1 on β-catenin transcription activity*. SW480 cells were transiently transfected with vector (pcDNA), PKD1 (pcDNA-PKD1) or PKD1-KD (kinase dead pcDNA-PKD1-K618W) along with reporter luciferase construct (a TCF-promoter-luciferase construct or a mutant TCF-promoter luciferase construct) and control plasmid expressing *Renilla* luciferase gene. The cells were harvested after 48h and assayed for firefly luciferase and normalized to control *Renilla* luciferase to measure β-catenin transcription activity, which was expressed as a ratio of TCF-promoter-luciferase activity/mutant TCF-promoter luciferase activity. The activity of control cells was set as 100%. PKD1 overexpression significantly decreased the β-catenin transcription activity, while overexpression of the kinase dead PKD1 released this inhibition, indicating the requirement of active PKD1 molecules for modulating β-catenin transcription activity. **(B)** *Site-specific expression of PKD1 in SW480 cells*. PKD1 overexpression was targeted to the nucleus or the membrane by transient transfection of SW480 cells with nuclear targeted PKD1 (GFP-NLS-PKD1) or membrane targeted PKD1 (Mem-PKD1-GFP). The cells were fixed and imaged for PKD1 overexpression (green) using confocal microscopy. The nuclei (blue) were counter-stained using DAPI. Nuclear targeted PKD1 (top row) was predominantly localized to the nucleus, while membrane targeted PKD1 (bottom row) was primarily localized on the cell membrane. Original magnification 1000X. **(C)**. *Effect of site-specific expression of PKD1 on β-catenin transcription activity*. SW480 cells were transiently transfected with vector (pEGFP), PKD1 (pEGFP-PKD1), nuclear targeted PKD1 (GFP-NLS-PKD1) or membrane targeted PKD1 (Mem-PKD1-GFP) along with reporter luciferase construct and control *Renilla* luciferase plasmid. After 48h, the cells were lysed and assayed for β-catenin co-transcription activity as mentioned above. Nuclear targeted PKD1 most effectively inhibited β-catenin transcription activity, while membrane localization of PKD1 released this inhibition, suggesting the need for nuclear PKD1 to inhibit β-catenin transcription activity. **(D)** *Expression of nuclear β-catenin*. Nuclear extract from cells overexpressing control vector, PKD1-GFP, and NLS-PKD1 were resolved on gel and immune-blotted for β-catenin and Histone H1 (internal control). Overexpression of nuclear PKD1 decreased nuclear β-catenin levels the most compared to control lysates.

The inhibition of β-catenin transcription activity was also confirmed using another independent construct. The PKD1 gene (pcDNA-PKD1) or control plasmid (pcDNA) was transiently overexpressed along with the luciferase reporter construct and evaluated for its effect on β-catenin transcription activity. As expected, PKD1 overexpression significantly inhibited β-catenin transcription activity (Figure 3A). In order to investigate if the kinase activity of PKD1 is necessary for the suppression of nuclear β-catenin transcription activity, we overexpressed a kinase dead mutant of PKD1 using a kinase-dead construct (pcDNA-PKD1-K618W) and analyzed the effect on β-catenin transcription activity. Interestingly, the kinase dead mutant of PKD1 failed to inhibit nuclear β-catenin transcriptional activity compared to vector control. In fact, an enhancement of β-catenin activity was observed in kinase dead mutant PKD1 overexpressing cells. This probably occurred due to a dominant-negative role in inhibiting the intrinsic functions of wild type PKD1 for this kinase dead mutant.

### Nuclear-targeted PKD1 more efficiently attenuates nuclear β-catenin transcription activity

PKD1 is primarily present in the cytoplasm, with a small amounts being present in the Golgi complex, the mitochondria, the nucleus and on the inner side of the cell membrane. To examine if nuclear PKD1 is required for the repression of nuclear β-catenin transcriptional activity, nucleus targeted PKD1-GFP construct (GFP-NLS-PKD1) and membrane targeted PKD1-GFP construct (Mem-PKD1-GFP) were overexpressed in SW480 cells. The site specific overexpression of PKD1 was confirmed by confocal microscopy (Figure 3B). Cells overexpressing PKD1 with a nuclear localization signal (GFP-NLS-PKD1) revealed exogenous PKD1 expression primarily in the nucleus (as seen by the green and cyan color in the overlay image of GFP-NLS-PKD1 (green) and nuclear signal DAPI (blue) (Figure 3B, top row). Cells overexpressing PKD1 with a membrane localization signal, however, revealed PKD1 expression primarily on the cell membrane (Figure 3B, bottom row). We then examined the effect of site-specific expression of PKD1 on β-catenin transcription activity. While PKD1 overexpression inhibited β-catenin transcription activity, the overexpression of nuclear-targeted PKD1 (GFP-NLS-PKD1) further enhanced the suppression of β-catenin transcription activity (Figure 3C). In contrast, the overexpression of membrane targeted PKD1 (Mem-PKD1-GFP) failed to suppress β-catenin transcription activity in the SW480 cells. To further confirm these findings, we prepared nuclear lysate from SW480 cells overexpressing compartment targeted PKD1 and immunoblotted for nuclear β-catenin and internal control Histone H1 (Figure 3D). While GFP overexpression did not cause any change in the expression of nuclear β-catenin, the overexpression of PKD1 or nuclear targeted PKD1 (GFP-NLS-PKD1) considerably decreased nuclear β-catenin levels (Figure 3D). The overexpression of nuclear targeted PKD1 (GFP-NLS-PKD1) caused the highest reduction in nuclear β-catenin levels in the SW480 cells compared to overexpression of either PKD1 or GFP. These results suggest a critical role for enzymatically active and nuclear localized PKD1 for the suppression of nuclear β-catenin transcription activity by lowering the levels of nuclear β-catenin within the cells.

### PKD1 overexpression enhances membrane localization of β-catenin

**Figure 4:**
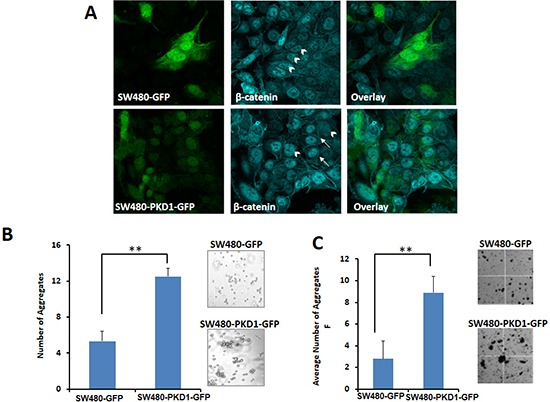
PKD1 overexpression enhances membrane localization of β-catenin and decreases nuclear β-catenin transcription activity **(A)** *β-catenin staining*. SW480 cells overexpressing PKD1 or GFP were seeded in chamber slides for 24h. The cells were fixed and processed for immunostaining using anti-β-catenin antibody. Representative confocal images of cells are shown for SW480-PKD1-GFP or SW480-GFP (green) and β-catenin (cyan) staining. The control cells (SW480-GFP) predominantly exhibited nuclear localization of β-catenin (arrow head) with very low membrane staining. However, PKD1 overexpressing cells showed relatively enhanced membrane localization of β-catenin (white arrows) along with nuclear localization. Original magnification 600X. **(B)** *Hanging drop cell-aggregation assay*. Equal volume of freshly trypsinized cells was spotted on the lid of a petri-dish and incubated under moist conditions for 24h. The aggregates formed were counted and photographed. PKD1 overexpression enhanced cell-cell aggregation, compared to control cells. Representative images of the cell-cell aggregates are also shown. Mean ± SE; n=3; **p<0.05. **(C)** *Cell-aggregation assay*. Freshly trypsinized SW480-PKD1-GFP or SW480-GFP cells were incubated in the presence of 2.5mM CaCl_2_ under mild shaking conditions to facilitate aggregate formation. The numbers of aggregates formed after 7h of incubation were enumerated, imaged and graphed. Representative images of the cell aggregation assay are also shown. PKD1 overexpression enhanced cell-cell aggregation, compared to control cells. Mean ± SD; n=2; **p<0.05

In addition to its role in signaling as a transcription factor, β-catenin plays a vital role in cell adhesion. It interacts with E-cadherin to form the cell-surface adhesion complex and enhances cell-cell adhesion [21]. Since the overexpression of PKD1 regulated the sub-cellular localization of β-catenin and decreased nuclear β-catenin expression, we then examined if PKD1 overexpression affects the expression of β-catenin on the cell membrane. Actively growing SW480-PKD1-GFP or SW480-GFP cells were fixed, stained using anti-β-catenin antibody and subjected to confocal microscopic analysis (Figure 4A). The expression of β-catenin was primarily nuclear (arrowheads) in the control cells, as is the case in the parent cell line. However, overexpression of PKD1 substantially enhanced the membrane localization of β-catenin compared to control cells (white arrows). A functional output of enhanced membrane localization of β-catenin might result in increased cell-cell adhesion. Therefore, to examine the functional consequence of enhanced membrane localization of β-catenin, the PKD1 overexpressing cells and control cells were subjected to two types of aggregation assays (Figure 4B and C). In the hanging-drop aggregation assay, the cells were trypsinized, spotted on the inner lid of a petri dish and incubated in an inverted position to form aggregates. The numbers of aggregates formed were examined after 24h (Figure 4B). Cells overexpressing PKD1 formed significantly higher numbers of aggregates (at least 3 fold) compared to control cells. Similar results were also observed in a second independent aggregation assay, wherein cells trypsinized under mild conditions were subjected to aggregate formation by incubating under gentle rocking conditions for 7h. PKD1 overexpressing cells formed markedly larger and a higher number of aggregates compared to control cells (Figure 4C).

### PKD1 overexpression suppresses cell motility

**Figure 5:**
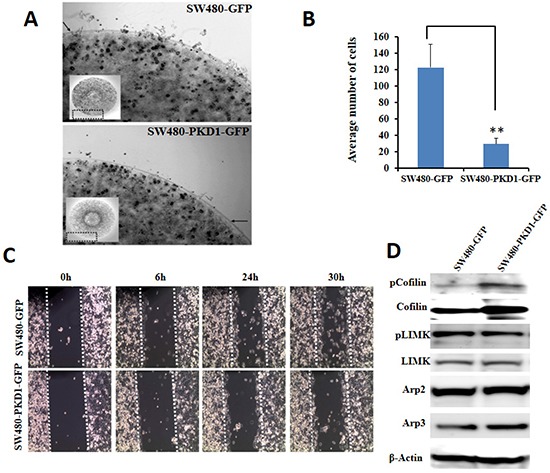
Overexpression of PKD1 inhibits cellular motility **(A)**. *Cell migration assay*. SW480 cells overexpressing PKD1 or GFP were mixed with agarose and equal volume was placed on fibrinonectin/BSA coated plates. Representative images of an agarose bead edge with motile cells are shown. The inset shows the corresponding whole agarose bead. PKD1 overexpression decreased the motility of SW480 cells. **(B)** *Quantitative analysis of cell migration assay*. The number of cells that migrated from the agarose bead was counted and plotted. PKD1 overexpression significantly inhibited migration of SW480 cells. Mean ± SE; n=3; **p<0.05. **(C)** *Scratch assay*. Confluent growth of SW480-PKD1-GFP or SW480-GFP cells was ‘wounded’ by a scratch using a micropipette tip. The ‘wound’ was periodically monitored for ‘wound healing’ and photographed. PKD1 overexpression decreased the motility of SW480 cells compared to vector control. **(D)** *Immunoblot analysis*. Representative blots of whole cell lysates from SW480-PKD1-GFP and control SW480-GFP cells were probed for proteins regulating cellular motility. PKD1 overexpression enhanced the expression levels of inactive phospho-cofilin and cofilin.

PKD1 plays a significant role in regulating cellular motility [18, 27, 28]. PKD1 has been shown to inhibit cellular motility by interacting with proteins at the leading edge of the motile cells. It negatively regulates cellular motility in part by indirectly maintaining the depolymerizing factor cofilin in its inactive phosphorylated form. PKD1 achieves this by enhancing cofilin phosphorylation (through PAK4-LIMK pathway) or by inhibiting its de-phosphorylation (through the direct phosphorylation and inhibition of SS1L phosphatase function) to shift the equilibrium towards maintaining the phosphorylated and inactive form of cofilin [14, 18]. Therefore, we evaluated the effect of PKD1 overexpression on motility of SW480 colon cancer cells using the agarose bead assay. In this test, the SW480-PKD1-GFP or SW480-GFP cells were embedded within agarose beads and spotted on cell culture plates pre-coated with fibrinonectin to examine the ability of cells to escape the beads and migrate on the surface of the plate (Figure 5A). PKD1 overexpression inhibited the motility of colon cancer cells compared to control SW480-GFP cells (Figure 5B). The ability of PKD1 to inhibit motility of SW480 colon cancer cells was also confirmed using a wound-healing assay (Figure 5C). A wound (or scratch) was created using the pointed edge of a sterile pipette tip on the surface of confluent plates of SW480-PKD1-GFP or SW480-GFP cells and the plates were examined at regular intervals to document and evaluate wound healing (or gap closure) by motile cells. SW480-GFP cells more effectively closed the gap/wound compared to PKD1 overexpressing cells (SW480-PKD1-GFP). In order to examine the molecular mechanisms regulating the cellular motility of the PKD1 overexpressing colon cancer cells, total protein lysates from SW480-PKD1-GFP or SW480-GFP cells were immunoblotted and examined for the expression of various motility related proteins (Figure 5D). We observed enhanced expression and phosphorylation of cofilin in PKD1 overexpressing cells compared to control cells. Little to no change was observed either in the expression or phosphorylation of other proteins involved in actin remodeling, including LIMK, Arp2 and Arp3. Thus PKD1 overexpression appeared to inhibit cellular motility partly by inhibiting the activity of cofilin, a protein critical for depolymerization of filamentous actin filaments to generate new monomeric actin for formation/extension of actin fibers at the leading edge.

### PKD1 influences *in-vivo* colon tumorigenesis

**Figure 6:**
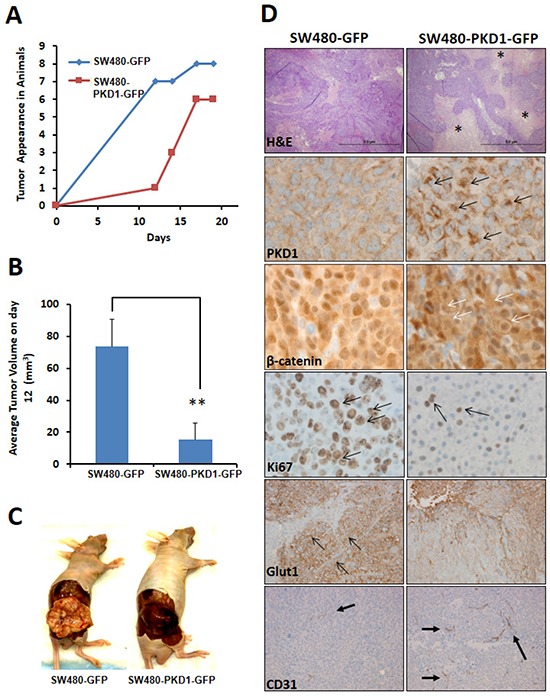
PKD1 overexpression delays tumor growth in xenograft mouse model **(A)** *Tumor appearance*. SW480-PKD1-GFP and SW480-GFP cells (5×10^6^) were injected into the hind flank of nude mice and tumor appearance was periodically monitored. The time required for visible tumor growth was graphed against the number of mice exhibiting the appearance of visible and measurable tumor (> 50mm^3^). PKD1 overexpression delayed tumor formation compared to GFP control cells. (n=8 mice/group). **(B)** *Tumor volume*. The average volume of the tumors formed by SW480-PKD1-GFP and SW480-GFP cells was measured on day 12 and plotted. The PKD1 overexpressing cells formed significantly smaller tumors by day 12 than control GFP cells. **(C)**. *Tumor in nude mice*. Representative photographs of nude mice showing tumors developed from SW480-PKD1-GFP or control SW480-GFP colon cancer cells. The tumors formed by SW480-GFP cells were nodulated, flat, and lighter in appearance. In contrast, the tumors formed by SW480-PKD1-GFP cells were smooth, round, and dark in appearance. **(D)** *Immuno-histochemistry of tumor tissues*. Paraffin embedded tumor xenografts were sectioned and immunohistochemically stained for necrosis (H and E), PKD1 overexpression, β-catenin localization, Ki67 (a marker for cell proliferation), Glut1 (a marker for hypoxia) and CD31 (marker for cellular vasculature). Compared to control tumors, the tumors formed by PKD1 overexpressing cells showed enhanced necrosis (necrotic regions are indicated by _*_), higher PKD1 staining (black arrows), elevated membrane localization of β-catenin (white arrows) and decreased Ki67 staining (arrows) that indicates lower cell proliferation. PKD1 overexpressing tumors also exhibited lower Glut1 staining and higher CD31 staining. This indicates that PKD1 overexpression decreased hypoxic conditions and increased vasculature in the tumor compared to GFP control tumors.

To investigate the tumor suppressor potential of PKD1 in colon carcinogenesis *in-vivo*, we examined the tumor growth pattern of PKD1 overexpressing cells in a xenograft mouse model. Equal number of SW480 cells overexpressing PKD1 or control vector (GFP) were subcutaneously (sc) injected into the hind flank of nude mice for tumor formation (n=8 per group). The mice were periodically examined for tumor appearance and tumor growth was monitored by calculating the volume of the tumor. By the 12^th^ day of injection, visible tumors could be seen and measured in most control animals. However, PKD1 overexpression in SW480 cells delayed the tumor appearance in nude mice compared to control SW480 cells overexpressing empty vector (Figure 6A). Analysis of the time taken for visible tumor formation (volume of 50mm^3^ or more) in each animal clearly showed a delay in tumor appearance in the PKD1 overexpressing cells compared to control group (Figure 6A). In addition, PKD1 overexpression also significantly decreased the average tumor size (volume) in nude mice compared to the tumor formed by control GFP overexpressing cells (Figure 6B). Intriguingly, the shape and overall appearance of tumors formed by the control SW480-GFP cells and the PKD1 overexpressing SW480-PKD1-GFP cells were considerably different. The tumors formed by control SW480-GFP cells appeared flat, nodulated, light pink/white in appearance and displayed well-formed blood vessels on the tumor surface. On the other hand, the tumors formed by PKD1 overexpressing cells appeared round, smooth and very dark in appearance (Figure 6C). The dark appearance of the tissue prompted us to examine the tissue for necrosis and vascularization. Therefore, the tumors were fixed, paraffin embedded and sliced into 5μM sections. These sections were stained with H&E to detect necrosis and also immunostained with PKD1 and β-catenin antibodies to confirm the presence of PKD1 overexpression and to analyze for change in the subcellular localization of the β-catenin. Additionally, we performed immunostaining for CD31 to detect vasculature in tumors. Interestingly, tumors formed by PKD1 overexpressing cells exhibited a higher degree of necrosis compared to control tumors and also demonstrated higher expression of PKD1 (Figure 6D). Importantly, akin to *in-vitro* observations, PKD1 overexpressing cells revealed considerably higher levels of membrane β-catenin on the surface of the cells than the control tumors, strongly implicating to the role of overexpressed PKD1 in modulating the functions and subcellular localization of β-catenin *in-vivo*. Additionally, PKD1 overexpressing tumors displayed higher number of blood vessels and more branching than control tumors (Figure 6D). A consequence of higher vasculature is oxygenation of the tumors and accordingly a lower expression of Glut1, a marker for hypoxia. Therefore, these tumors were stained for Glut1 to verify the degree of hypoxia. Indeed, tumor tissues formed by PKD1 overexpressing cells showed much lower expression of Glut1 compared to control tumors. These results indicate that PKD1 overexpressing cells not only initially delayed the appearance of tumor, but they eventually formed relatively smaller and necrotic tumors compared to control cells, strongly supporting a tumor suppressor function for PKD1 in colon cancer. These data also suggest a role of PKD1 in tumor necrosis.

## DISCUSSION

The highly conserved β-catenin protein regulates cell proliferation, polarity and cellular fate determination and thereby plays a prominent role in tightly controlling multiple processes including embryogenesis and cellular homeostasis [5, 6, 29]. Therefore, the dysregulation of β–catenin functions leads to major problems in cellular proliferation and differentiation, eventually resulting in the development of a number of cancers [29]. The role of β-catenin is especially well documented in colon cancer [2]. In fact, mutation in the Wnt/β-catenin signaling pathway is responsible for over 80% of all types of colon cancer [2]. Therefore, an in-depth understanding of the regulation and modulation of the Wnt/β-catenin signaling pathway will aid in developing effective treatment strategies for colon cancer. This study aims to delineate a molecular association between PKD1 and β-catenin to develop an effective therapeutic strategy for colon cancer.

The serine-threonine kinase PKD1 has previously been shown to bind, phosphorylate and regulate the functions of β-catenin. The phosphorylation of β-catenin by PKD1 results in translocating β-catenin out of the nucleus and suppressing the transcription functions of the nuclear β-catenin [10, 11, 19, 30]. This decreased transcriptional activity of β-catenin results in reduced expression of oncogenes like c-Myc and cyclin D1 and ultimately the inhibition of cell proliferation and cancerous properties of the cells [19]. Additionally, the activation of PKD1 using natural compounds like curcumin or Bryostatin-1, in concurrence with the observed tumor suppressor function for PKD1, also leads to decreased nuclear β-catenin functions and lower cell proliferation in prostate cancer cells [19, 31]. PKD1 has also been attributed to possess a tumor suppressor function in breast cancer. The overexpression of PKD1 in breast cancer cells inhibited multiple metalloproteinases, suppressed cellular motility, prevented epithelial to mesenchymal transition and modulated the tumor microenvironment leading to the suppression of tumor growth/progression [13, 32-34]. Although PKD1 has been shown to be downregulated in many cancers, including prostate and breast cancers, its expression patterns and role in colon cancer has never been investigated.

Herein, for the first time, we sought to investigate the role of PKD1 in colon cancer. We examined its function in the regulation of β-catenin signaling pathway since this is one of the main pathways which usually operates aberrantly in colon cancer. Our expression analysis revealed a conspicuous decrease in PKD1 levels in higher grade colon cancer samples compared to normal colon and early grade samples (Figure 1). In our *in-vitro* studies, the overexpression of PKD1 significantly decreased the nuclear β-catenin levels and β-catenin transcriptional activity and thus reduced expression of the pro-carcinogenic downstream targets like cyclin D1. It was an intriguing observation to find that the nuclear β-catenin transcriptional activity was predominantly influenced by nucleus targeted PKD1. Further investigations revealed that this subcellular modulation of β-catenin results in enhanced membrane localization of β-catenin and thereby, increases cell-cell adhesion which is severely compromised in cancer cells. A similar function of PKD1 was demonstrated in prostate cancer cells [19, 21]. This eventually resulted in decreasing cell proliferation and clonogenic potential in colon cancer cells (Figure 2). These anti-proliferative results are consistent with the findings in prostate and breast cancer, wherein the overexpression or activation of PKD1 suppressed cancerous phenotype of the cells [13, 19, 20, 31, 33]. Herein, we have also shown that the attenuation of nuclear β-catenin functions is accomplished by enzymatically active PKD1 that is primarily present in the nucleus (Figure 3). Our results also revealed that PKD1 overexpression reduced the cellular motility of colon cancer cells by inhibiting the functions of the cofilin protein that are critical for the actin remodeling and cellular motility. Thus, overexpression of PKD1 in colon cancer cells not only enhanced cell-cell interaction, but also inhibited cell motility (Figure 4, 5). Our studies are in accordance with other studies which also suggest the role of PKD1 in suppression of cellular motility of cancer cells [14, 18, 27, 28, 35]. Additionally, the disruption of β-catenin/TCF complex formation was found on PKD1 expression that is important to regulate the proliferation and progression of colon cancer cells. This also controls the resulting activation of the genetic program in colorectal transformation process [9].

Interestingly, our *in-vitro* results of PKD1 were recapitulated in xenograft *in-vivo* animal experiments. The *in-vivo* investigation using a xenograft mouse model revealed that PKD1 overexpression delayed the time of tumor appearance and tumor development compared to control GFP overexpressing cells. The detection of lower nuclear β-catenin levels, higher membrane localization of β-catenin and reduced staining of Ki67 (a marker for cell proliferation) was observed in the tumors formed by PKD1 overexpressing cells compared to control GFP overexpressing tumors. This data strongly suggests that PKD1 overexpression attenuates nuclear β-catenin functions and thus suppresses tumor growth. Interestingly, the tumors formed by PKD1 overexpressing cells revealed higher necrotic cell death compared to control tumors. Previous work has suggested a role for PKD1 in inducing programmed necrotic cell death and autophagy [36, 37]. Upon activation by oxidative stress, activated PKD1 in turn may activate the JNK pathway resulting in the programmed necrosis [36, 38]. Our results provide the first *in-vivo* evidence implicating a role of PKD1 in necrosis. However, the higher degree of tumor necrosis observed was not due to hypoxia within the tumor. The hypoxic core in tumors is a common occurrence due to dense and rapid cellular growth of the cancer cells without the simultaneous development of blood capillaries, leading to necrotic cell death. Our results suggest that the tumors formed by control GFP overexpressing cells were highly hypoxic compared to the tumors formed by PKD1 overexpressing cells. This observation is significant, since hypoxia within tumors leads to the activation of the transcription factor, hypoxia inducing factor (HIF), that eventually induces the synthesis of proteins resulting in highly aggressive tumor metastasis [39, 40]. A possible reason for the lower hypoxia might be due to sufficient blood vessel growth. Our results suggest that PKD1 overexpression enhances the formation of blood vessels. The ability of PKD1 to decrease tumor hypoxia and enhance tumor vasculature suggest that PKD1 can improve delivery of cancer drug(s) in tumors. Previous reports implicate a role for PKD1 in VEGF induced angiogenesis through the modulation of class II histone deacetylases [41-43]. PKD1 plays a critical downstream role in VEGF-mediated activation of downstream targets enhancing blood vessel formation [43]. Due to these molecular alterations, PKD1 overexpression causes conspicuous change in tumor morphology, structure and histo-architecture. The change in tumor shape might be a result of modulation of not only the protein involved in adherens junctions but also other cell-cell binding and cell-stroma binding factors. Important roles of PKD1 in regulating E-cadherin and β-catenin mediated adherens junctions have previously been shown in prostate cancer cells [19, 21, 44]. Together, these results suggest that the overexpression or activation of PKD1 in tumors enhanced tumor cell death and lowered hypoxia within the tumors. Further analysis of patient sample and PKD1 activators in animal mouse models will yield important information on the role of PKD1 in tumor metastasis and the development of effective treatment strategies.

In conclusion, our studies revealed a novel tumor suppressor function for PKD1 in colon cancer. We have found a correlation of PKD1 downregulation with the aberrant expression and nuclear localization of β-catenin in human colon cancer tissues. *In vitro* investigation revealed that PKD1 directly interacts with β-catenin and attenuates β-catenin transcriptional activity by decreasing nuclear β-catenin levels. Moreover, functional assays including PKD1 overexpression in colon cancer cells inhibited cellular motility and enhanced cell-cell adhesion. The *in-vivo* experiments suggests that PKD1 overexpression delayed tumor appearance and formed smaller tumors by modulating β-catenin functions in colon cancer. Based on these results, we propose that PKD1 may act as a tumor suppressor in colon cancer by modulating the nuclear β-catenin/Wnt signaling. Therefore, strategies for the up-regulation of PKD1 expression levels and/or activation in colon cancer cells are desired to modulate the nuclear β-catenin/Wnt signaling in colon cancer. Thus, the identification of drug molecules that induce PKD1 overexpression/activation may be important for the development of novel therapeutic modalities to inhibit tumorigenesis and colon cancer progression.

## METHODS

### Cell lines and other materials

Colon cancer cells SW480 and SW48 were purchased from ATCC (ATCC, Manassas, Virginia). The cell lines LoVo, HT29 and T-84 were kindly provided by Prof. Keith Johnson (University of Nebraska Medical Center, Omaha, Nebraska). These cell lines were propagated in high glucose DMEM media supplemented with glutamine, 100 mM sodium pyruvate, 10% fetal bovine serum (FBS) and 1X antibiotic and antimycotic solution. The media components were purchased from Hyclone (Hyclone Laboratories, South Logan, UT), unless mentioned otherwise. OPTI-MEM reduced serum growth media was purchased from Invitrogen (Life Technologies, Carlsbad, CA). All other chemicals were purchased from Sigma (Sigma-Aldrich, St. Louis, MO) unless mentioned otherwise.

### Antibodies

Rabbit polyclonal PKD1 antibody (C-20) and Histone H1 were procured from Santa Cruz Biotechnologies (Santa Cruz, CA). Rabbit monoclonal PKD1, cofilin, phospho-cofilin, Arp3, LIMK, phospho-LIMK and Cyclin D1 were purchased from Cell Signaling Technologies (Danvers, MA). Ki67, CD31, Glut1 and β-actin antibodies were purchased from Sigma-Aldrich. The mouse monoclonal β-catenin antibody is a generous gift of Dr. Keith Johnson (University of Nebraska Medical Center, Omaha, Nebraska), the use and specificity of which has been previously described [45]. The HRP conjugated secondary antibodies were purchased from Promega, (Madison, WI) and fluorescence tagged anti-mouse secondary antibodies were purchased from Jackson ImmunoResearch Laboratories (Westgrove, PA).

### Immunohistochemical (IHC) staining of tissue samples

The tissue microarray slides (AccuMax, ISU Abxis Co., Ltd, Seoul, Korea) and the slides from colon cancer xenograft mouse tumor were stained using heat-induced antigen retrieval immunohistochemistry techniques with the Vector ABC kit (Vector Laboratories, Burlingame, CA) or Biocare kit (Biocare Medical, Concord, CA) and analyzed as previously described [31]. Briefly, the slides containing the tumor tissues were deparaffinized, rehydrated, treated with 0.3% hydrogen peroxide or peroxidazed solution (Biocare Medical) and processed for antigen retrieval using heat-induced technique. After blocking nonspecific binding with background sniper (Biocare Medical), the tissues were incubated with primary antibodies (PKD1 (1:100), β-catenin (1:100), Ki67 (1:25), CD31 (1:25) or Glut1 (1:100). The final detection for the expression of the specific protein was carried out by using either fluorescently labeled secondary antibodies (1:150) or chromogenic dyes. For the detection of protein using fluorescent antibodies, the slides were incubated in the dark with fluorescently labelled secondary antibodies (1:150), washed and mounted using Vectamount (Vector Laboratories). For the final detection of protein using chromogenic dyes 3,3′-diaminobenzidine (DAB) or Vulcan red, the samples were processed using MACH 4 Universal HRP Polymer detection kit (Biocare Medical) according to manufacturer's instructions and developed using DAB (DAB substrate kit, Vector Laboratories). These slides were counter-stained using hematoxylin and mounted using Vectamount (Vector Laboratories). The slides stained with fluorescent secondary antibodies were processed for laser scanning confocal microscopy with an Olympus Fluoview FV1000 confocal microscope (Olympus Corporation, Tokyo, Japan), while the chromogenically stained slides were visualized using an Olympus BX 41 Microscope (Olympus Corporation).

### Analysis of IHC samples

Quantitative examination of the TMA samples were independently analyzed by two pathologists at the Sanford School of Medicine and the mean composite score (MCS) was calculated as previously mentioned [46, 47]. The samples were evaluated for staining intensity on a scale of 0 to 4 (0 for no immunostaining, and 4 for very high staining). In addition, the samples were also analyzed for the extent of staining and expressed as percentage of cancer cells that had stained for the protein of interest. This percentage of stained cells was also scored on the scale of 0 to 4 (0 for less than 5% staining, 1 for 5-25%, 2 for 26-50%, 3 for 51-75% and 4 for >75% positively stained cells). The MCS for each sample was calculated by multiplying the percentage of cancer cells positively stained with the intensity of staining (range of 0-16).

### Western blotting

Actively growing colon cancer cells were used for immunoblot analysis as described earlier [31]. Briefly, cells (70-80% confluent) were washed with ice-cold phosphate buffer saline (PBS) and lysed in 2X SDS lysis buffer. Equivalent amounts of protein samples were electrophoretically resolved on 4-20% SDS-PAGE gels, blotted onto PVDF membrane (Bio-Rad Laboratories, Hercules, CA), blocked with 5% bovine serum albumin (BSA; 5 ml for one hour) and probed for various proteins using specific primary antibodies. The western blots were incubated with HRP-labeled secondary antibody and the protein bands were developed using Lumi-Light Plus chemi-luminescent reagent (Roche, Indianapolis, IN).

### Immunofluorescence

SW480 or SW48 cells expressing various GFP tagged constructs (1.5×10^5^) were seeded in a 4-well chamber slides (Thermo Scientific Nunc, Waltham, MA) for 48 h and processed for immunofluorescence as previously described [31]. In brief, the cells were fixed in 2% paraformaldehyde (PFA) for 15 min, mounted in Vectashield (Vector Laboratories) and processed for laser scanning confocal microscopy with an Olympus Fluoview FV1000 confocal microscope (Olympus Corporation). In order to detect the localization of β-catenin in these cells, following PFA fixation the cells were permeabilized for 5 min with chilled methanol, incubated with anti-β-catenin primary antibody (1:10) for 1 h, and detected by incubating with Cy3 labeled secondary antibodies for 1 h. The slide was mounted in Vectashield mounting media (Vector Laboratories) and processed for laser confocal microscopy.

### Transfection and generation of stable cell line

The pEGFP vector containing PKD1, GFP-NLS-PKD1, Mem-PKD1-GFP and PKD1-KD (kinase-dead) were kind gift from Drs K.C. Balaji (Wake Forest School of Medicine, Salem, NC) and Cheng Du (University of Massachusetts Medical School, Worcester, MA). Colon cancer cell lines (SW480 or SW48) were transfected with pEGFP vector or pEGFP vector containing PKD1 gene or GFP-NLS-PKD1 gene (PKD1 gene tagged to a nuclear localization signal), or Mem-PKD1-GFP gene (PKD1 gene tagged to a membrane localization signal) or PKD1-KD (PKD1 gene with a point mutation at the 618 residue that renders it kinase dead (PKD1 K618W)) using Lipofectamine2000 (Invitrogen) in a serum free media, as previously described [47]. After 6 hours of transfection, the media was replaced with 10% serum containing media. The transfected cells were propagated in the presence of a selection agent (500 μg/mL of G418; Invitrogen) and used for experiments within 2-3 passages following transfection. The SW480 cells were also transfected with pcDNA3.1 or PKD1 gene cloned in pcDNA3.1 vector as mentioned above. In order to isolate SW480 stable cell lines overexpressing PKD1-GFP or control GFP, actively growing SW480 cells (80% confluent) were transfected with PKD1 gene cloned in pEGFP.C1 vector or empty vector using Lipofectamine2000 (Invitrogen) and propagated in the presence of 500 μg/mL of G418 (Invitrogen) selection agent for the selection of stably transfected cells as previously described [47]. A pool of stably transfected SW480 cells overexpressing PKD1-GFP (referred to as SW480-PKD1-GFP) or control SW480 cells stably overexpressing GFP (referred to as SW480-GFP) were enriched for stably transfected cells by subjecting these cells to fluorescence assisted cell sorting (FACS). The enriched pool of cells, maintained under constant G418 selection, were expanded and frozen into multiple aliquots of stock. To maintain authenticity of the stable cell lines, the cells were always maintained in the presence of G418 selection agent and used for 30-35 passages, after which a fresh cell line stock was thawed and used. As wild-type and vector control cells did not show any significant differences, and in order to avoid redundancy, the results are primarily shown for vector control.

### Cell Proliferation

Cell proliferation was determined by either using CellTiter-Glo Luminescent cell viability assay (Promega) or by manual counting method. The measurement using the CellTiter-Glo Luminescent cell viability assay was carried out according to manufacturer's instructions. Briefly, 5×10^3^ cells of SW480-GFP or SW480-PKD1-GFP were plated in 96-well plates and incubated for 48h in a humidified incubator at 37°C/5% CO_2_. Cell proliferation was assessed by measuring the amount of ATP in the cells using CellTiter-Glo Reagent. The determination of cell proliferation by manual counting method was carried out as previously described [47]. Briefly, cells (2×10^4^) were seeded in 6-well plates in triplicate and after varying periods of time (24, 48, 72, and 96 h) the cells were harvested and manually counted using a hemocytometer.

### Anchorage dependent and anchorage independent colony formation assay

Both colony formation assays were performed as described earlier [31, 48]. To determine the anchorage dependent colony formation, cells (2×10^3^) were plated in 100mm cell culture dishes for 12 days. The colonies formed were fixed with methanol, stained with hematoxylin and number of visible colonies were manually counted and plotted as previously described [31]. The anchorage independent colony formation assay was carried out in 6-well plates as previously described [31]. A bottom 0.6% agarose layer was first cast in the plates. Following solidification, the top 0.35% agarose layer containing cells (4×10^4^) was cast. Following 14 days of incubation in 4 ml media per well, the colonies were either directly imaged or stained with 0.05% crystal violet and imaged using a phase contrast microscope. Average numbers of colonies were counted from five independent areas and plotted.

### Aggregation assay

The aggregation assay was performed as described earlier [31]. In brief, actively growing cells were trypsinized (0.01% trypsin-EDTA) and washed with PBS containing 5mM CaCl_2_. 3×10^6^ cells (1×10^6^ cells/ml) were resuspended in 15ml polystyrene tubes in DMEM containing 5mM CaCl_2_, incubated for 7h at 37°C under mild mixing/shaking conditions and imaged for number of aggregates formed using a phase contrast microscope. A second type of aggregation assay was also performed as preciously described [19]. Actively growing cells were trypsinized, resuspended at 2×10^4^ cell/ml and 25 μl drops were spotted onto the inner side of a 25 mm petri plate lid. The lid was carefully inverted over the petri plate containing 2ml PBS and incubated for 24h in a humidified incubator at 37°C in the presence of 5% CO_2_. The cells were gently resuspended and imaged under microscope for aggregate formation.

### Cell motility assay

The scratch assay for determining cell motility was performed as previously described [21, 49]. Briefly, cells (1×10^6^cells/plate) were cultured in 35mm plates until confluent and using the sharp side of a 20μl sterile tip, the confluent cell culture was scratched to generate a wound/gap and incubated at 37°C in the presence of 5% CO_2_. The scratch was periodically imaged using an EVOS microscope (Advanced Microscope Group, Bothell, WA) at varying time intervals. A second assay to determine cellular motility using the agarose beads (agarose bead motility assay) was carried out as described earlier [47]. Equal volumes of cells (1×10^7^ cells/ml) and 0.7% low melting agarose were mixed and 25 μl drops were spotted onto 6-well plates pretreated with Fibrinonectin (15μg/ml) and BSA (10μg/ml). Following gelling, the beads were incubated in 2ml media at 37°C and photographed at regular time intervals using a phase contrast microscope. The average number of motile cells that had escaped out of each bead was counted and plotted.

### β-catenin/TCF Luciferase Reporter assay

The reporter constructs were a generous gift from Dr. R. Moon (University of Washington, Seattle, WA). The luciferase reporter assay to determine β-catenin/TCF transcription activity was carried out as previously described [19, 31]. Briefly, actively growing stable cell lines of SW480 overexpressing either PKD1 or GFP cells (1.5×10^5^ cells/well) were plated in triplicate in 12-well plates for 24-36h and transiently co-transfected with TCF-firefly luciferase reporter construct (pTOP-FLASH) and *Renilla* luciferase internal control plasmid (pRL-TK) (Promega). Non-specific/background transcription activity was determined by transiently transfecting the control wells with mutant TCF promoter construct (pFOP-FLASH) and *Renilla* luciferase construct (pRL-TK). After 24h, the cell lysates were prepared and assayed for firefly luciferase and *Renilla* luciferase activity using Dual Glo reagents (Promega) according to the manufacturer's instructions and the luciferase signal was measured in a GloMax 96 Microplate Luminometer (Promega). The β-catenin/TCF transcription activity was determined by normalizing the firefly luciferase activity to that of *Renilla* luciferase activity and calculating the ratio of TOP-FLASH signal to FOP-FLASH signal.

Transient transfection of the colon cancer cell lines was also used to examine the effect of the various construct of PKD1 on β-catenin transcription activity. Briefly, actively growing cells (1.5×10^5^ cells/well) were plated as mentioned above and transiently co-transfected with TCF-firefly luciferase reporter construct (pTOP-FLASH) and *Renilla* luciferase construct along with one of the various PKD1 constructs or control plasmid. Non-specific/background transcription activity was determined by transiently transfecting the control wells with mutant TCF promoter sites (pFOP-FLASH) and *Renilla* luciferase construct (pRL-TK) and the corresponding PKD1 constructs or control plasmid. The cell lysates were prepared and assayed as mentioned above.

### Tumor xenograft model

Six-week-old male athymic nude (nu/nu) mice (Charles River Laboratories, Wilmington, MA) were used to generate colon cancer xenografts as described earlier [31]. The mice were maintained in a pathogen-free environment and all procedures were carried out as approved by the Sanford Research/University of South Dakota Institutional Animal Care and Use Committee. Briefly, SW480 cells overexpressing PKD1 or GFP (5×10^6^ cells/100μl/per mouse) were mixed with 100 μl Matrigel (BD Biosciences, Sparks, MD) and injected subcutaneously (sc) into the flank of the left hind limb. The animals were periodically monitored for tumor development and the tumor volume was measured from day 12 after injection using a digital Vernier caliper. The tumor volume was calculated using the ellipsoid volume formula: tumor volume (mm^3^) = *π*/6 × *L* × *W* × *H*, wherein *L* is length, *W* is width, and *H* is height. The tumor growth was regularly monitored till either the end of the study or until the tumor burden reached a volume of 700mm^3^. The mice were sacrificed, the tumors fixed in formalin, embedded in paraffin, and sliced into 5μM sections for further processing and analysis.

### Statistical analyses

Student's t test was used for analysis of statistical significance and the significance was determined using a paired t-test. A *p* value of < 0.05 was considered significant.

## SUPPLEMENTARY FIGURES


