# Supplementary material for: Protein Kinase D1 attenuates tumorigenesis in colon cancer by modulating β-catenin/T cell factor activity
Source: Oncotarget. 2014 Aug 4;5(16):6867–84. doi: 10.18632/oncotarget.2277 (PMC4196169; doi:10.18632/oncotarget.2277)
Supplement: Supplementary file 1 [file oncotarget-05-6867-s001.pdf]

SUPPLEMENTARY FIGURES

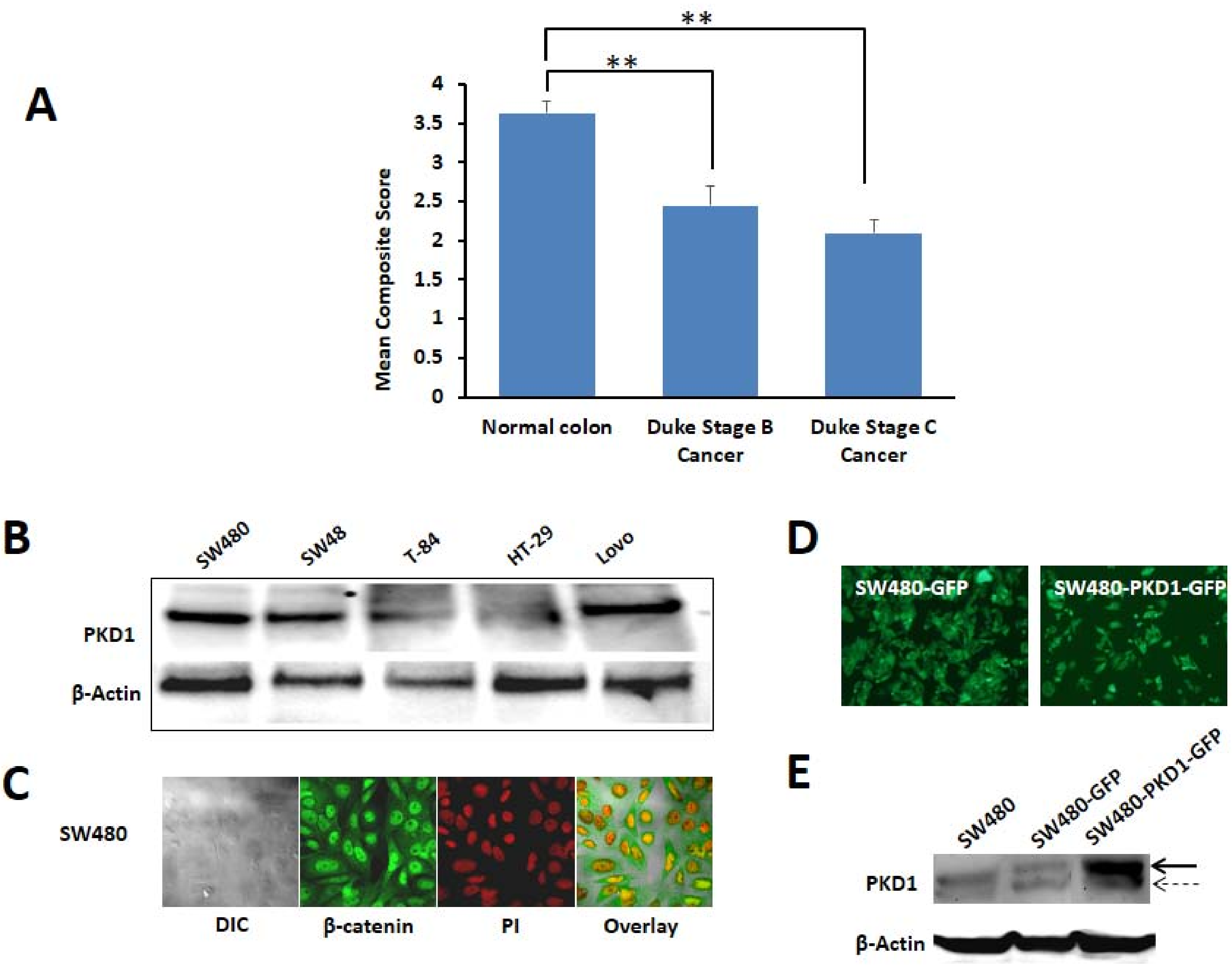

Supplementary Figure S1: PKD1 and β-catenin expression in tissue microarray slides and SW480 colon cancer cells. **(A)** *Quantitative analysis of PKD1 staining in Tissue microarray:* The colon cancer TMA samples stained for PKD1 (red) were evaluated by two pathologists for the intensity and extent of staining. The mean composite score (MCS) for each TMA sample was calculated as detailed in the Methods Section and the MCS was graphed with respect to the Duke's stages of colon cancer. A substantial and significant decrease in the expression of PKD1 was detected in Duke's B and C stages of colon cancer compared to control tissues. **(B)** *PKD1 expression in colon cancer cell lines.* Representative western blot of whole cell lysates isolated from various colon cancer cell lines and probed for PKD1 expression. β-actin was used as loading control. **(C)** *β-catenin in SW480 cells.* SW480 cells were seeded in chamber slides for 24h. The cells were fixed and processed for immunostaining using anti-β-catenin antibody (green), and the nuclei (red) was counter-stained using propidium Iodite (PI). The overlay image shows distinct localization of β-catenin in the nucleus (yellow). Original magnification 400X. **(D)** *PKD1 expression in stable cell lines.* Fluorescent and phase contrast microscopic images of stably transfected SW480 cells overexpressing either GFP tagged PKD1 or GFP. Original magnification 100X. **(E)** *Western blot analysis of stable cell lines.* Cell lysates from SW480, SW480-GFP and SW480-PKD1-GFP were resolved on SDS-PAGE, blotted on PVDF membrane and probed for PKD1 expression using anti-PKD1 antibody. β-actin was used as loading control. The intrinsic PKD1 (dotted arrow) and exogenous overexpressed GFP tagged PKD1 (solid arrow) are indicated in the blot.

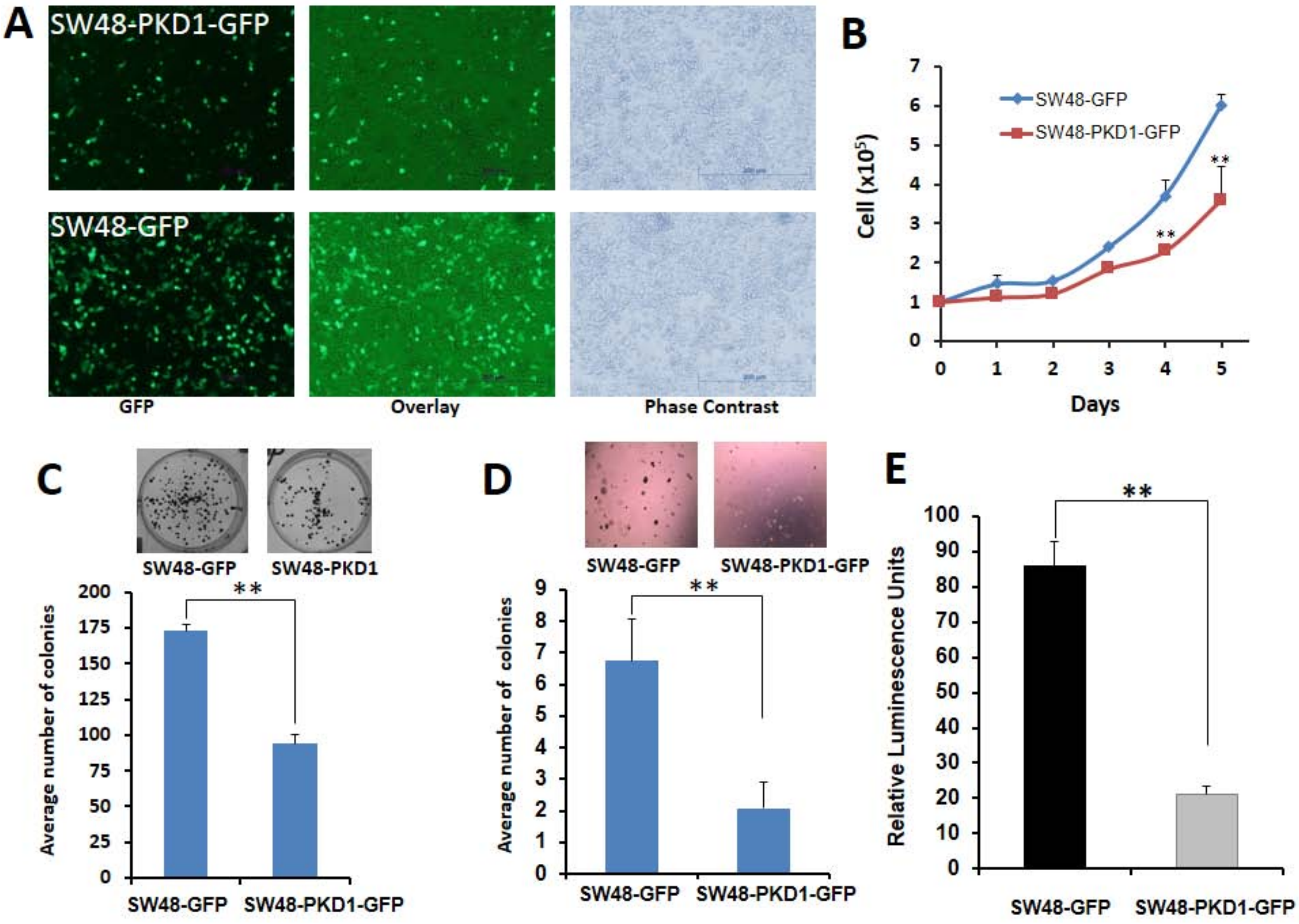

Supplementary Figure S2: Effect of PKD1 overexpression in SW48 colon cancer cells. **(A)** *Overexpression of PKD1 in SW48 cells*. Fluorescent and phase contrast microscopic images of SW48 cells overexpressing either PKD1 or GFP are shown. Original magnification 100X. **(B)** *Cell proliferation of SW48 cells*. Equal numbers of SW48-GFP and SW48-PKD1-GFP cells were plated in multiple cell culture plates. The cells were harvested for five consecutive days, enumerated and graphed. PKD1 overexpression significantly decreased cell proliferation compared to control cells. Mean ± SE; n=3; **$p<0.05$. **(C)** *Anchorage dependent colony formation*. SW48 cells overexpressing either PKD1 or GFP vector ($2x10^3$) were plated in 100mm dishes for 12 days and the number of colonies formed was counted and graphed. Representative images of colonies are shown below the graph. PKD1 overexpression suppressed anchorage dependent colony formation in SW48 cells. Mean ± SE; n=3; **$p<0.05$. **(D)** *Anchorage independent colony formation.* SW48-GFP and SW48-PKD1-GFP cells ($4x10^4$) were seeded in soft agar and grown for 14 days and the number of colonies formed was enumerated and plotted. Representative images of colonies are shown above the graph. PKD1 overexpression decreased anchorage independent colony formation in SW48 cells. Mean ± SE; n=3; **$p<0.05$. **(E)** *β-catenin transcription activity.* SW48 cells were transiently transfected with vector (pEGFP) or PKD1 (pEGFP-PKD1) along with reporter luciferase construct and an internal control plasmid. The cells were harvested after 48h and assayed to measure β-catenin co-transcription activity as mentioned earlier. PKD1 overexpression significantly decreased β-catenin transcription activity by over 75%.
